# Influence of Tohoku-Pacific ocean earthquake on headache cases in the affected area

**DOI:** 10.1186/1129-2377-14-S1-P22

**Published:** 2013-02-21

**Authors:** Y Matsumori, S Fujiwara

**Affiliations:** 1 Department of Headache Medicine, Kohnan Hospital, Japan; 2 Department of Neurosurgery, Kohnan Hospital, Japan

## Introduction

On March 11, 2011, Tohoku-Pacific Ocean Earthquake with a magnitude of 9.0 occurred in East Japan, affecting a part of our hospital located in Sendai causing serious damage. The tsunami struck 4 km away from our hospital. This disaster caused considerable damage to various lifelines including food and medical supplies. Some transportation networks were also paralyzed for several weeks. Although there were such limitations, our hospital continued to conduct medical examination of patients including outpatients.

## Purpose

The aim of this study was to examine how the disaster influenced the headache (HA) medicine at our hospital. Method: We compared the situation of outpatient consultation for HA cases, severity of pain, impact on daily life, and types of HA, which included migraine, tension-type HA (TTH), cluster HA (CH), and medication-overuse HA (MOH).

## Results

The number of outpatient HA cases before the disaster was 9.5 persons/day, and the occurrence rates of the HA types were 62.2% for migraine, 38.6% for TTH, 3.0% for CH, and 10.6% for MOH. The number of HA cases decreased remarkably after the disaster (1.6 persons/day) in March, after which it increased gradually (8.1 persons/day) in July. After the disaster, although the severity of pain did not change, the impact on daily life because of migraine became significantly worse (p < 0.001). The occurrence rate of migraine increased and that of TTH decreased significantly (p < 0.001). The occurrence rate of MOH increased slightly, and no change was seen in the occurrence rate of CH.

## Conclusion

After the disaster, although the HA outpatient consultation rate fell evidently, the occurrence rate of severe migraine increased at our hospital. Although it was difficult to visit a hospital due to difficulties as a result of the disaster, an HA patient particularly with the high impact on daily life visited hospital to seek help.
